# Why Do People With Self-Control Forgive Others Easily? The Role of Rumination and Anger

**DOI:** 10.3389/fpsyg.2020.00129

**Published:** 2020-02-20

**Authors:** Fanchang Kong, Haibo Zhang, Haishuo Xia, Bo Huang, Jingkuan Qin, Yan Zhang, Xiaojun Sun, Zongkui Zhou

**Affiliations:** ^1^Key Laboratory of Adolescent Cyberpsychology and Behavior, Ministry of Education, Wuhan, China; ^2^School of Psychology, Central China Normal University, Wuhan, China; ^3^Faculty of Psychology, Southwest University, Beibei, China; ^4^School of Educational Science, Huazhong University of Science and Technology, Wuhan, China

**Keywords:** self-control, rumination, anger, trait forgiveness, serial mediation effect

## Abstract

Previous research shows that self-control predicts forgiveness, but few studies have investigated the internal mechanism of this link. The current study explored the effects of rumination and anger on the relationship between self-control and forgiveness. A total of 580 college students recruited from three universities in Wuhan completed the self-control, rumination, anger, and trait forgiveness scales. Results showed that self-control was positively correlated with forgiveness (*r* = 0.34, *p* < 0.001). Rumination (*β* = 0.08, *p* < 0.05) and anger (*β* = 0.13, *p* < 0.05) mediate the relationship between self-control and forgiveness. Moreover, a serial mediation effect of rumination and anger was observed between self-control and trait forgiveness (*β* = 0.02, *p* < 0.05). These findings suggest that self-control may impair individuals’ rumination. Moreover, less rumination may restrain anger and consequently increase forgiveness.

## Introduction

Connecting with others and maintaining harmonious interpersonal relationships is necessary within society. However, interpersonal relationships are often disturbed by certain offenses, which may result in the breakdown of relationships between the victim and the offender; opportunities for downstream gains from such relationships may also be lost (McCullough et al., 2013). The drawbacks of ending a relationship normally motivate victims to choose forgiveness to maintain the original state of the relationship (McCullough et al., 2010). At the dispositional level, individuals differ much in their willingness to forgive, and trait forgiveness has been conceptualized as a basis for responses to specific transgressions (Roberts, 1995). According to the interpersonal variable model, personalities are important factors of trait forgiveness (Koutsos et al., 2008). People with a high level of agreeableness or empathy often show forgiveness in a specific relationship (McCullough and Hoyt, 2002; Giammarco and Vernon, 2014).

Self-control is the ability to monitor and regulate behavior to achieve long-term goals (Balliet, 2010; Pronk et al., 2010). People who have poor self-control may perform unconstrained impulsive actions to serve immediate urges, desires, and emotions (Hagger et al., 2010). Moreover, self-control plays a role in social relationships (McCullough et al., 2013).

### Relationship Between Self-Control and Trait Forgiveness

McCullough et al. (1998) have argued that individuals undergo complex motivation in the case of an offense. On the one hand, offense behavior, as a stress factor, instinctively triggers victims’ negative interpersonal motivations (e.g., avoidance or revenge) toward offenders (Finkel and Campbell, 2001). On the other hand, unforgiveness means losing the possibility to gain potential benefits from these relationships; victims may regain the prosocial motivation to get along with offenders (McCullough et al., 2010). Prosocial motivation and negative interpersonal motivation are tightly related; forgiveness occurs when prosocial motivation is stronger than negative interpersonal motivation (McCullough et al., 1998). According to interdependence theory, self-control plays a role in motivation transformation when individuals are in mixed motivation states; a person with a high level of self-control can easily transfer negative interpersonal motivation into prosocial motivation (Righetti et al., 2013). According to the compensation model, trait self-control is positively related to forgiveness even in the absence of concern for the well-being of others (Balliet et al., 2011). Beyond these observations, the result of a mate analysis shows that individuals with a high level of self-control often have a high tendency to forgive others (Burnette et al., 2014). In other words, self-control can positively predict trait forgiveness (Balliet et al., 2011; Yin, 2016). On the basis of previous findings, we suggest the following hypothesis: *Self-control positively predicts trait forgiveness* (H1).

### Mediation Effect of Rumination Between Self-Control and Trait Forgiveness

Rumination, as a negative cognition process, is a strategy used when dealing with a negative experience; this process involves repetitive and passive focus on one’s negative experiences (Denson et al., 2011). Rumination often makes people feel uncomfortable, thus increasing their tendency toward aggression (Denson et al., 2011). When people experience an offense, they recall details repeatedly and think about the negative consequences of the offense. Rumination involves central executive functions (Smith and Alloy, 2009), and self-control is closely related to the advanced cognitive activity and can therefore suppress rumination through central executive functions (Hofmann et al., 2012). Meanwhile, a study has shown that self-control is negatively related to rumination (Denson et al., 2011), thus indicating that self-control may predict one’s rumination.

The relationship between rumination and forgiveness has been investigated several times. Lucas et al. (2010) has found that rumination is negatively related with forgiveness. Furthermore, Berkowitz (2012) has reported that rumination makes victims experience offended feelings repeatedly, which ultimately hampers forgiveness. Moreover, researchers have indicated that rumination can negatively predict forgiveness, and the possibility of forgiveness can be increased by reducing rumination (Fatfouta, 2015; Witvliet et al., 2015). In other words, rumination may predict trait forgiveness negatively. Meanwhile, rumination is considered one of the most important factors in interpersonal forgiveness among college students (Zhang et al., 2017). A review study has noted that cognitive factors, such as rumination, may be a considerable predictor for forgiveness (Ma and Zheng, 2012). Furthermore, a recent study has shown that rumination partly mediates the relationship between self-control and trait forgiveness, suggesting that self-control may increase forgiveness by decreasing the level of rumination (Xia et al., 2017). On the basis of previous findings, we formulate the following hypothesis: *Self-control positively predicts trait forgiveness through rumination* (H2).

### Mediation Effect of Anger Between Self-Control and Trait Forgiveness

Except for cognitive processes, self-control may also influence emotional regulation (Hofmann et al., 2012; Watkins et al., 2013). Watkins et al. (2013) have found that a high level of self-control can help individuals perform effective emotional management and ensure that emotions can be expressed appropriately. Empirical studies have suggested that self-control can effectively reduce anger (Chapman et al., 2006). Another study has shown that high school students with a high level of self-control tend to express less anger (Hamarta et al., 2015). In sum, self-control may predict anger negatively.

Moreover, anger is a key factor that prevents trait forgiveness. Numerous offenses often trigger fierce anger within victims, thus making forgiveness difficult. First, anger reduction from the event that triggered anger to the current state is positively related to forgiveness (Konstam et al., 2001). Then, a cross-cultural investigation has found a negative association between anger and forgiveness in both U.S. and Chinese culture (Zhang et al., 2015). Furthermore, a meta-analysis has suggested that people with a high level of anger are less likely to grant forgiveness than those with a low level of anger (Fehr et al., 2010). In other words, anger and forgiveness are negatively correlated. To explore the causal relationship between anger and forgiveness, a longitudinal study has found that the level of anger can negatively predict the level of forgiveness (McCullough et al., 2007). Therefore, we formulate the following hypothesis: *Self-control positively predicts trait forgiveness through the mediation of anger* (H3).

### Multiple Mediation Effect of Rumination and Anger Between Self-Control and Trait Forgiveness

Rumination is an important risk factor for anger. The ABC theory of emotion demonstrates that cognition is the direct cause of emotion (Ziegler, 2001). We have deduced that rumination can predict anger, and numerous empirical studies have investigated the link between them. Berkowitz (2012) has stated that rumination, which is conceptualized as the repeated recall of aggressive behaviors, can activate the feelings of being offended and eventually trigger anger. Bushman (2002) has also found that rumination can instigate anger. Runions (2013) has shown that the anger of victims toward offenders can be effectively reduced by decreasing rumination. Thus, rumination may be a negative predictor of anger. Furthermore, a recent study among Chinese college students has shown that rumination and anger are positively correlated with each other, and anger and rumination are both negatively associated with forgiveness (Wu et al., 2019). However, no study has examined the role of anger and rumination simultaneously in the relationship between self-control and forgiveness. To explore the underlying mechanism of the influence of self-control on forgiveness clearly, we propose the following hypothesis: *Self-control positively predicts trait forgiveness through the multiple mediations of rumination and anger* (H4).

**Figure 1:**
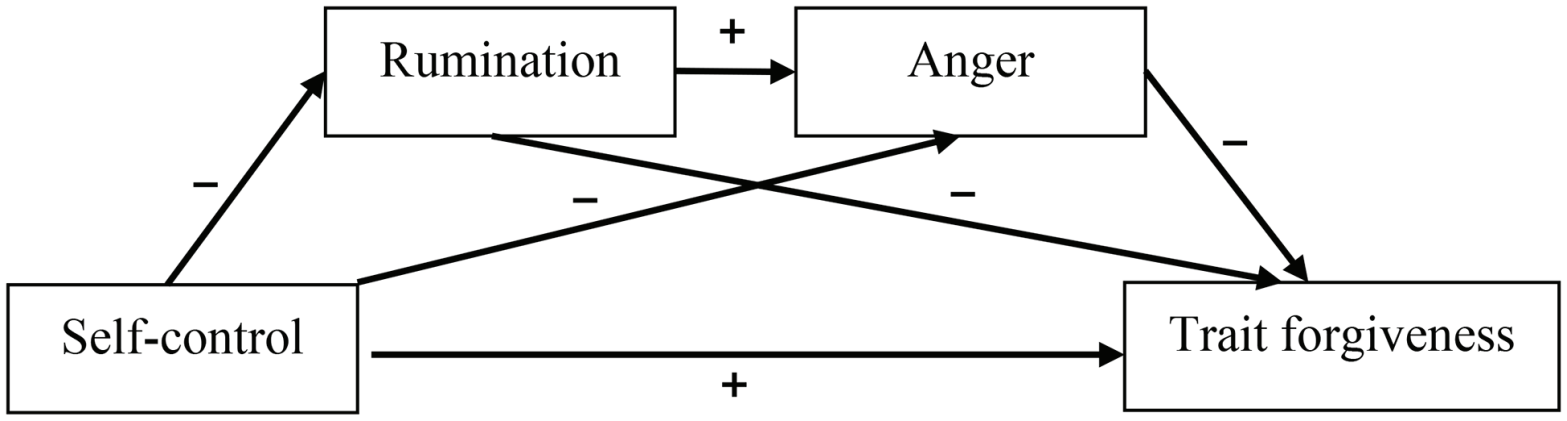
Conceptual model.

The present study explores how self-control influences trait forgiveness by building multiple mediation models (Figure 1). This study will provide new evidence to determine how self-control influences forgiveness and to guide the intervention and treatment of forgiveness behaviors.

## Method

### Participants

A total of 580 students were randomly recruited from three universities in Wuhan, Hubei province. The final valid sample size was 573, which comprised 284 females, 276 males, and 13 unreported students. Seven participants were excluded because the completion of their questionnaire was lower than two-thirds of the total items. The participants’ age ranged from 16 to 36, and the mean age was 22.26 years (SD = 4.01).

### Measures

#### Self-Control Scale

Self-control was measured with the 19-item self-control scale, which was revised from the Chinese samples by Tan and Guo (2008). The items were rated by a five-point scale ranging from 1 (*totally disagree*) to 5 (*totally agree*). One example was the statement, “I can resist the temptation easily.” The total score ranged from 19 to 95. High scores indicated great self-control. The scale was a reliable and valid measurement of self-control (Tan and Guo, 2008). In this study, the Cronbach’s *α* was 0.86, and the fit indices of confirmatory factor analysis were shown as follows: *χ*^2^/df = 2.35, NFI = 0.90, CFI = 0.94, RMSEA = 0.05.

#### Rumination Scale

The Chinese version of the seven-item rumination scale was developed by Wang (2006), which included items such as “Sad things in the past always come to my mind, which makes it hard to fall asleep.” The items were rated by a 5-point scale ranging from 1 (*totally disagree*) to 5 (*totally agree*). The total score ranged from 7 to 35. Higher scores indicated more rumination. The scale showed excellent reliability and validity (Wang, 2006). In the present research, the Cronbach’s *α* was 0.87, and the fit indices of confirmatory factor analysis were shown as follows: *χ*^2^/df = 3.77, NFI = 0.98, CFI = 0.98, RMSEA = 0.07.

#### Anger Scale

This scale was the sub-scale of the State-Trait Anger Expression Inventory (STAXI) (Spielberger, 1999), which was used to measure an individual’s level of anger after an offense. The Chinese version of the sub-scale was revised by Liu and Gao (2012), which included six items. Each item was rated by a five-point scale ranging from 1 (*totally disagree*) to 5 (*totally agree*). The total score ranged from 6 to 30. Higher scores indicated more anger. An example of the questions included “I will feel angry if I am behind schedule because of others’ mistakes.” In this study, the Cronbach’s *α* was 0.81, and the fit indices of confirmatory factor analysis were shown as follows: *χ*^2^/df = 3.50, NFI = 0.96, CFI = 0.97, RMSEA = 0.07.

#### Trait Forgiveness Scale

The scale included the Heartland Forgiveness Scale (HFS)-Self sub-scale with items such as “I always feel regretful about my mistakes” and HFS-Other sub-scale with items like “In most cases, I can forgive others for their faults” (Thompson et al., 2005). The two sub-scales had 12 items each, but only the HFS-Other was employed for studying individuals’ forgiveness tendency toward others. The items were rated by a five-point scale ranging from 1 (*totally disagree*) to 5 (*totally agree*). The total score ranged from 12 to 60. Higher scores indicated higher trait forgiveness. In the current study, the Cronbach’s *α* was 0.80. The fit indices of confirmatory factor analysis were shown as follows: *χ*^2^/df = 4.26, NFI = 0.90, CFI = 0.92, RMSEA = 0.07.

### Procedure and Data Processing

This study was approved by the Human Research Ethics Committee, School of Psychology in XXX University and all participants signed an informed consent form before their inclusion in the formal research. The participants were students recruited randomly from three universities in Wuhan, China. These participants were supposed to read the instructions carefully before completing the scale. The data were collected in person, and each participant was given a large pack of tissue worth 10 RMB after the investigation. The missing data were replaced by the population mean value because less than 0.1% of the total data were missing. We conducted descriptive analysis, correlation analysis, and multiple mediation analysis on self-control, rumination, anger, and trait forgiveness.

## Results

### Control and Test of Common Method Bias

Using self-report to collect data might result in common method bias, so certain actions were taken (Zhou and Long, 2004). Participants answered the questions completely and anonymously, and some items were reverse coded. Furthermore, Harman’s single-factor test was used to test the common method bias when the data were collected. The results showed that 11 factors had an eigenvalue that was more than 1. The first factor accounted for 20.25% of the total variance, which was less than the critical standard 40% (Zhou and Long, 2004). Therefore, no significant common method bias was observed.

### Descriptive Statistics and Correlations

After controlling the effects of age and sex, the results showed significant correlations between every two variables. The absolute values of the correlation coefficients were between 0.30 and 0.55 (Table 1).

**Table 1:** Descriptive statistics and correlations among variables.

	*M* ± SD	1	2	3	4
1. Self-control	59.01 ± 10.30	—			
2. Rumination	18.69 ± 5.96	−0.43^***^	—		
3. Anger	22.21 ± 4.79	−0.51^***^	0.36^***^	—	
4. Trait forgiveness	39.67 ± 6.77	0.34^***^	−0.35^***^	−0.43^***^	—

*** *p < 0.001 (all two-tailed test)*.

### Multiple Mediation of Rumination and Anger

**Figure 2:**
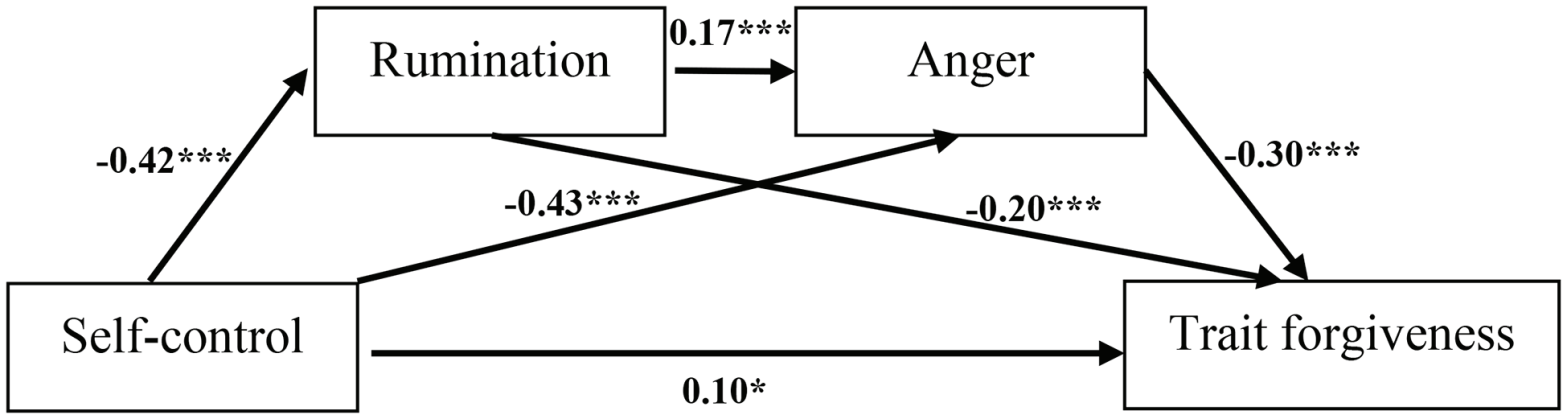
Multiple mediation model.

Bias-corrected percentile bootstrap method was used to test the mediation effect of rumination and anger (Hayes, 2013). The number of bootstrap samples was 5,000. The level of confidence for all confidence intervals in the output was 95%. Given that the CI did not contain 0, the mediation effect was significant. The results showed that after controlling gender and age, all the paths were significant in the overall model (Figure 2).

On the direct effect, trait forgiveness was predicted by self-control significantly. On the indirect effects, the total indirect effect was significant (Table 2). Specifically, rumination was the meditator [*β* = 0.08, *p* < 0.05, CI (0.05, 0.13)] in the path of self-control → rumination → trait forgiveness (Indirect effect 1). Anger was the meditator variable [*β* = 0.13, *p* < 0.05, CI (0.09, 0.18)] in the path of self-control → anger → trait forgiveness (Indirect effect 2). A serial mediation effect of rumination and anger was observed [*β* = 0.02, *p* < 0.05, CI (0.01, 0.04)] in the path of self-control → rumination → anger → trait forgiveness (Indirect effect 3).

**Table 2:** Indirect effects of self-control and trait forgiveness.

	*β*	Boot SE	Boot LLCI	Boot ULCI	Ratio of total effects
Total indirect effect	0.23	0.03	0.18	0.29	69.70%	
Indirect effect 1	0.08	0.02	0.05	0.13	24.24%	
Indirect effect 2	0.13	0.02	0.09	0.18	39.40%	
Indirect effect 3	0.02	0.01	0.01	0.04	6.06%	

*β refers to standardization effect size*.

## Discussion

### Relationship Between Self-Control and Trait Forgiveness

The study finds that self-control can positively predict trait forgiveness, which supports H1. This finding is consistent with that of previous research (Balliet et al., 2011; Yin, 2016; Xia et al., 2017). According to interdependence theory, a low level of self-control makes individuals susceptible to negative interpersonal motivation and causes them to act impulsively (Righetti et al., 2013). Another explanation is that enhancing self-control will help improve problem-solving strategies, such as negotiation instead of revenge, and, eventually, the act of forgiveness.

### Mediation Effects of Rumination and Anger in the Relationship of Self-Control and Trait Forgiveness

The results show that self-control can positively predict trait forgiveness through rumination, which supports H2. This finding has also been proven by Xia et al. (2017), indicating that rumination mediates the relationship between self-control and forgiveness. Self-control, as a type of basic human ability, has a broad influence on cognition processes (Ma and Zheng, 2012). People with high level of self-control can efficiently decrease rumination to improve the tendency of forgiveness.

Moreover, the results show that self-control can predict trait forgiveness through anger, which supports H3. This finding is also consistent with Ma and Zheng’s (2012) view that emotional factor plays a role in the relationship between self-control and trait forgiveness. Previous research has found that anger is a negative factor for forgiveness (Fehr et al., 2010; Zhang et al., 2015), and reducing anger promotes forgiveness (Konstam et al., 2001). The present study explores the role of anger in the relationship between self-control and forgiveness.

In addition, self-control influences trait forgiveness through the multiple mediation effect of rumination and anger, which supports H4. Berkowitz (2012) has shown that rumination allows individuals to experience high levels of anger and found that the act of forgiveness is challenged by activating negative information about offenses. Meanwhile, individuals with high level of self-control are likely to restrain rumination (Denson et al., 2011; Xia et al., 2017), have less anger, and can eventually forgive others. As mentioned above, rumination is a kind of cognition process, and anger is a specific emotion. Therefore, according to the ABC theory, we can speculate that rumination may be a cognition process that can lead to anger. Self-control subsequently predicts trait forgiveness through the mediators of rumination and anger in the current study. Moreover, these findings elaborate the theory of Ma and Zheng (2012) and enrich our understanding of mediators of rumination and anger in the relationship between self-control and trait forgiveness among Chinese college students. However, the perspectives about the direction from rumination to anger vary. Some studies have suggested that rumination is indirectly associated with forgiveness through anger (Bushman, 2002; Ray et al., 2008). Other studies have suggested that anger may improve the level of rumination, particularly anger rumination, which is a kind of rumination that recall past anger experiences. Thus, future studies should further examine the relationship, mainly the direction, of rumination and anger through a longitudinal design. The present study is the first to investigate the inner mechanism underlying self-control and forgiveness in the Chinese context. A serial meditation model has been conducted to enrich the theory of forgiveness behaviors and provide important suggestions for practice in the intervention of forgiveness. In practice, victims can reduce rumination by improving the level of self-control. Then, they may feel fewer negative emotions and eventually increase their willingness to forgive offenders.

### Limitations

This study has a few limitations. First, the causal relationship of self-control and forgiveness has not been tested due to the cross-sectional research design. Future research should use longitudinal design or a three-wave cross-lagged design to examine the causal relationship of self-control and trait forgiveness. Second, the act of forgiveness varies in different backgrounds and events in which offense is inflicted. Thus, these factors should be considered in future studies to investigate the relationship between self-control and trait forgiveness. Third, this study has focused only on dispositional states of self-control, anger, rumination, and forgiveness. Finally, the participants of this study are mainly college students. Therefore, the results are not extensively representative. In the future, replicating the present study with a large sample group can address the issue of generalizability.

## Conclusion

Self-control positively predicts trait forgiveness directly and through the chain mediation of rumination and anger indirectly.

## Data Availability Statement

The raw data supporting the conclusions of this manuscript will be made available by the authors, without undue reservation, to any qualified researcher.

## Ethics Statement

The studies involving human participants were reviewed and approved by Central China Normal University Human Ethics Committee. The patients/participants provided their written informed consent to participate in this study.

## Author Contributions

FK designed the research and revised the paper. HZ and HX collected and analyzed the data and wrote the paper. BH and JQ recruited participants and collected data. XS, ZZ, and YZ revised the paper. All authors agreed on the final version of the manuscript.

### Conflict of Interest

The authors declare that the research was conducted in the absence of any commercial or financial relationships that could be construed as a potential conflict of interest.
